# N^6^-methyladenosine (m^6^A) RNA modification as a metabolic switch between plant cell survival and death in leaf senescence

**DOI:** 10.3389/fpls.2022.1064131

**Published:** 2023-01-04

**Authors:** Elżbieta Rudy, Magda Grabsztunowicz, Magdalena Arasimowicz-Jelonek, Umesh Kumar Tanwar, Julia Maciorowska, Ewa Sobieszczuk-Nowicka

**Affiliations:** ^1^ Department of Plant Physiology, Faculty of Biology, Adam Mickiewicz University in Poznań, Uniwersytetu Poznańskiego 6, Poznań, Poland; ^2^ Department of Plant Ecophysiology, Faculty of Biology, Adam Mickiewicz University in Poznań, Uniwersytetu Poznańskiego 6, Poznań, Poland

**Keywords:** epitranscriptomics, m^6^A, leaf senescence, abiotic stress, crop improvement, barley, RNA modifications

## Abstract

Crop losses caused by climate change and various (a)biotic stressors negatively affect agriculture and crop production. Therefore, it is vital to develop a proper understanding of the complex response(s) to (a)biotic stresses and delineate them for each crop plant as a means to enable translational research. In plants, the improvement of crop quality by m^6^A editing is believed to be a promising strategy. As a reaction to environmental changes, m^6^A modification showed a high degree of sensitivity and complexity. We investigated differences in gene medleys between dark-induced leaf senescence (DILS) and developmental leaf senescence in barley, including *inter alia* RNA modifications active in DILS. The identified upregulated genes in DILS include RNA methyltransferases of different RNA types, embracing enzymes modifying mRNA, tRNA, and rRNA. We have defined a decisive moment in the DILS model which determines the point of no return, but the mechanism of its control is yet to be uncovered. This indicates the possibility of an unknown additional switch between cell survival and cell death. Discoveries of m^6^A RNA modification changes in certain RNA species in different stages of leaf senescence may uncover the role of such modifications in metabolic reprogramming. Nonetheless, there is no such data about the process of leaf senescence in plants. In this scope, the prospect of finding connections between the process of senescence and m^6^A modification of RNA in plants seems to be compelling.

## 1 Introduction

For the past 20 years, scientists have been building up a wealth of research that shows the ubiquity of both natural and artificially produced epigenetic variability, as well as how it can affect phenotypes (agronomic features) and significantly improve crops. One example is epitranscriptomics, defined as posttranscriptional modifications of RNA bases that alter gene expression without modifying the RNA sequence and regulate the stability and transport of the RNA.

**Figure 1:**
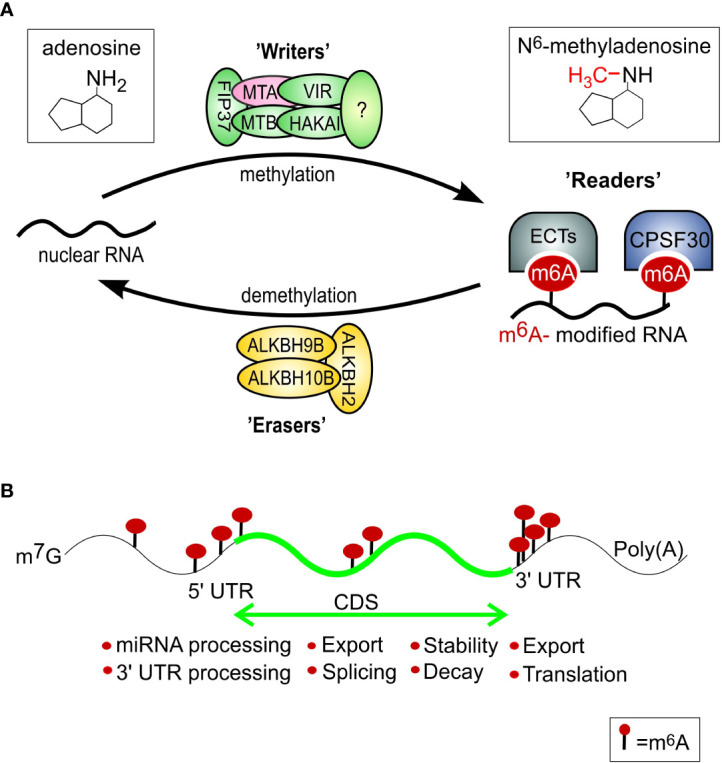
m^6^A mRNA modifications. **(A)** Regulation of mRNA m**^6^** A modification in plants through the action of a network of m^6^A writers (methyltransferase), erasers (demethylase), and reader proteins. The m^6^A writer complex consists of the proteins MTA, MTB, FIP37, VIRILIZER, and HAKAI. The m^6^A modifications can be removed by ALKBH2, ALKBH9B, and ALKBH10B proteins within the nucleus. The ECT2/3/4 and CPSF30 proteins serve as m^6^A readers that bind specifically to m^6^A sites and mediate distinct functions. The expression of subunit MTA (pink) was shown to be upregulated during DILS previously (Sobieszczuk- Nowicka et al., 2018). **(B)** Typical m^6^A distribution in regions of an mRNA and its readout affects mRNA fates, including trafficking, stability, decay, translation, and localization. Based on: Zheng et al., 2020; Sokpor et al., 2021; modified.

N^6^-methyladenosine (m^6^A) is the most frequently occurring and conserved internal modification of eukaryotic RNA (**Figure 1**). Furthermore, this modification is dynamic and reversible; hence, it has been proposed to be a key regulator for numerous physiological aspects of living organisms, among others growth, development, and response to stress (Zaccara et al., 2019; Shao et al., 2021; Zhou et al., 2022). Lately, several studies have reported that modification of m^6^A RNA is a key regulatory agent in aging and cell senescence in animals (Sun et al., 2021; Wu et al., 2022). Nonetheless, similar research has not been done on plants to date. Therefore, epigenetic control of the level of senescence-inducing signals associated with the overall environmental cues and the developmental program is possible.

When there is an instant need for a group of proteins to be expressed, ranging from tens to thousands, epigenetic modifications may enable a rapid response to signaling and stimuli. The regulation of gene expression by the genetic and epigenetic modifications, which may have heritable and nonheritable effects, may help the crop survive and/or improve. We must therefore expand our understanding of the fundamental molecular mechanisms underlying the epigenetic regulators in crops that control stress-induced senescence. A tool for further exploitation in the direction of sustainable agriculture may be provided by understanding the mechanism of epigenetic regulators and their regulatory networks in this process in crops. Finding the epigenetic regulatory aspects of senescence in crops may present a possible tool for increasing the quality and production of the crop as senescence occurs as an ordered process to demolish the vegetative tissues and divert nutrients to metabolic pathways for reproductive success.

This perspective article synthesizes the knowledge in the scope of epigenetic regulation of induced leaf senescence based on the modification of m^6^A RNA, as well as discusses the likelihood of applying this knowledge to enhance crop quality. In the article, the question is raised whether the m^6^A RNA modification changes in certain RNA species in different stages of leaf senescence may uncover the role of such modifications in the metabolic reprogramming of leaf cells between strategies of cell survival and cell death. We have defined a decisive moment in the dark-induced leaf senescence model (DILS) (Sobieszczuk-Nowicka et al., 2018) which determines the point of no return, but the mechanism of its control is unknown. It was revealed that in the barley crop model for early and late events of DILS there are differences in RNA modifications active in DILS in comparison with developmental leaf senescence. This hints at the possibility of a future discovery of an epigenetic-based switch between the survival and death of cells.

## 2 Senescence as a developmental stage that fosters phenotypic plasticity so that organisms can adapt to their environment under certain conditions

As per the current state of knowledge, senescence is a developmental stage that: (i) is an event of transient differentiation after growth; (ii) might or might not be followed by death; and (iii) is wholly dependent on the viability of cells and a particular gene expression (Thomas, 2013). Senescence in plants is a highly controlled and dynamic process that necessitates a global metabolic reprogramming effort aimed at organized resource breakdown and mobilization. It is a fundamental component of plant growth, crucial for maximizing resource use and encouraging phenotypic plasticity so that plants can adapt to their environment under specific conditions (Paluch-Lubawa et al., 2021 and references therein).

Plants’ growth is greatly supported by leaves, which are the best organs for using light energy and producing additional photosynthates with simultaneous minimization of total anabolic cost. When under stress, it may be advantageous for the plant if a leaf that is not photosynthetically active passes through senescence and makes its resources available to other organs. Senescence induction needs to be tightly managed in order to prevent activation that only occurs under short-term adverse situations. Light is essential for controlling the senescence of leaves, which is consistent with the significance of leaves in photosynthesis. Many plant species experience fast senescence as a result of light deprivation, especially when the plant is only partially impacted (reviewed by Liebsch and Keech, 2016).

Senescence brought on by darkness has become a reliable experimental model for research on the development of leaf senescence (Sobieszczuk-Nowicka et al., 2018). The study of leaf senescence is complicated by the absence of coordinated cell growth within a single leaf. As a result, induced senescence, like senescence brought on by darkness and controlling a synchronous process, has become relevant. Additionally, it removes deceptive characteristics associated with developing leaf senescence (DLS), such as shooting or flowering (reviewed by Paluch-Lubawa et al., 2021).

*Arabidopsis* is a very appealing model for the discovery and functional investigation of genes regulated by senescence due to the availability of its genomic resources (Buchanan-Wollaston et al., 2003; Breeze et al., 2011). Nonetheless, removing developing flowers and pods significantly lengthens the life of leaves in many plants, such as barley, whereas in *Arabidopsis*, male-sterile mutants or plants from which developing shoots have been removed do not prolong the life of leaves (Zimmermann et al., 2006). Research has found that the senescence process of *Arabidopsis* differs further from that of monocotyledonous plants. Cereal senescence is mostly controlled at the single-leaf level. Older leaf nutrients are remobilized for younger leaves, and then for the flag leaf, providing the grain with the nutrition it needs to grow. Cereal leaves have a meristem base, an older cell population at the leaf tip, and a concentration of younger cells at the leaf base. It is easier to distinguish the development of senescence due to the arrangement of the cells (Gregersen et al., 2008). These clear differences make it necessary to use cereal leaves as an additional model to the *Arabidopsis* for studies of senescence in cereal.

## 3 Understanding induced senescence and its economic importance; m^6^A RNA modification as a new player in the scope of regulators of gene expression

Understanding induced senescence is crucial for the economy since it can significantly reduce crop losses and limit the shelf life after harvest. Our knowledge of leaf senescence and its fundamental molecular regulation has advanced significantly in the last few decades. The senescence window concept, a theoretical model, explains how the ability to enter senescence is defined during leaf development, and how the length of senescence is governed by a combination of internal and external factors along with age (Jing et al., 2003). By emphasizing the many developmental stages, it will be feasible to use more targeted tactics to manage senescence.

It has been reported that active modifications of DNA and RNA commonly occur during DILS (but not DLS) events (Sobieszczuk-Nowicka et al., 2018). It is not unanticipated that epigenetic regulation is seemingly involved in senescence-related processes, because mechanisms such as methylation of DNA, rearrangement of ATP-dependent nucleosomes, and modification of post-translational histone have been proven to have key roles in regulating gene expression in stress resistance (reviewed by Chang et al., 2020), as well as plant growth and development (reviewed by Sun et al., 2021). The epigenetic mechanisms mentioned above are intertwined and often mutually dependent and they result in changes in chromatin status, switching from actively transcribed euchromatin to transcriptionally inactive heterochromatin and oppositely. Such a regulation level allows quick responses to stimuli when the expression of a gene group (ranging from tens to thousands) needs to be adjusted rapidly (Eriksson et al., 2020).

In the field of gene expression regulators, specifically RNA chemical changes, new players have recently surfaced. RNA modifications were first discussed in the 1950s (Davis & Allen, 1957; Cohn, 1959), but recently have they attracted more attention from researchers. This led to the “static” view of RNA’s involvement in cells being questioned (Gilbert, 1986). Currently, all living organisms have been found to have over 170 post-transcriptional RNA modifications (Williams et al., 2019), and they are stored in a number of databases, including the MODOMICS database. Since RNA modifications are one of the most evolutionary constant characteristics of RNAs (Li and Mason, 2014), they reveal a novel, complex layer of biological regulation called the epitranscriptome (Saletore et al., 2013).

In general, RNA modifications have been divided into reversible and non-reversible. The first group consists of well-characterized phenomena such as RNA editing and pseudouridylation (Meier et al., 2016; Romano et al., 2018), whereas the recent focus has been on reversible modifications including methylations of cytosine and adenosine (Romano et al., 2018). As it has been shown, RNA methylation affects several biological processes e.g. RNA stability and mRNA translation (Chmielowska-Bąk et al., 2019; Wang et al., 2022). One of them, N^6^-methyladenosine, is thought to be the most prevalent, reversible epitranscriptomic mark, functionally active in both animal and plant RNAs. It occurs when a methyl group is attached to the nitrogen-containing base at the sixth position of the adenine residue of RNA (**Figure 1**) (Batista et al., 2014; Shen et al., 2021). Current research reveals that one of the major processes in RNA post-transcriptional regulation, the m^6^A modification, controls RNA fate (Guo et al., 2022; Kumar and Mohapatra, 2021; Yin et al., 2022).

The dynamic interplay between RNA methyltransferases called ‘writers’ and RNA demethylase ‘erasers’ determine the effects and abundance of m^6^A on RNAs (Yu et al., 2021) (**Figure 1**). The third protein group, designed as ‘readers’, takes part in the recognition and processing of methylated RNAs (Meyer & Jaffrey, 2014). In animals, genes encoding m^6^A writer, reader, and eraser proteins (Yang et al., 2018) have been identified and shown to be vital for normal development, as well as adaptations to several environmental stresses (reviewed by Meyer & Jaffrey, 2014; Miryeganeh, 2021). Components of m^6^A modification have been present within the plant’s common ancestor since early plant evolution, and the protein domains associated with each of these components were highly conserved in pteridophytes, angiosperms, and algae (Dominissini et al., 2016). Besides the messenger RNAs (mRNAs), transfer RNAs (tRNAs), ribosomal RNAs (rRNAs), small non-coding RNAs, and long non-coding RNAs (lncRNAs) can be modified by m^6^A. These modifications regulate several angles of RNA processing, i.a. stability, alternative splicing, translation, export, and maturation of miRNA (Meyer et al., 2015; Yu et al., 2021). Comprehensive research on plants has shown that m^6^A modification is significant for meristem function (perhaps particularly for the differentiation step of stem cells), maintenance of circadian and seasonal plant rhythms, and late flowering (Sun et al, 2022a; Wang et al., 2021). Mutant plants lacking the genes coding for ‘writers’ of m^6^A modification are characterized by abnormal organ definition, loss of apical dominance, slower growth, and an increased number of trichome branches (Růžička et al., 2017; Arribas-Hernández et al., 2018). Additionally, the role of m^6^A in the selective stabilization of mRNAs in salt and osmotic stress has been reported for Arabidopsis (Anderson et al., 2018; Arribas-Hernández et al., 2018; Hu et al., 2021). Even with the recent progress in the field, the identity and function of most writers, readers, and erasers in plants are just beginning to be explored.

## 4 Discussion: Modification of m^6^A RNA and induced-senescence of barley leaf

We must further our understanding of the epigenetic regulators in crops during senescence brought on by stress and their basic molecular mechanism. Investigating *Arabidopsis* plants made it possible to present a theoretical model about how the potential to senesce is formed during leaf development and possibly how internal and external factors coincide with age to define the timing of senescence (Jing et al., 2003). Nonetheless, such a model seems not to be suitable to describe the complexity of senescence in all plant species. Among cereals, barley (*Hordeum vulgare*) is a model organism both genetically and genomically: it is characterized by a high degree of natural variation and by the adaptability to numerous different cultivation environments. Its simple diploid genome has already been sequenced (Beier et al., 2017) and the self-pollinating mating system, along with the availability of genetic and genomic resources, is the reason this plant is a reference model. Additionally, barley is one of the major and important crops because it ranks fourth among grain cereals (*Gramineae* species) following maize, wheat, and rice when it comes to global production (Giraldo et al., 2019). The volume of global production for the barley was estimated at 159.74 million metric tons in the 2020/2021 crop year (https://www.statista.com). As a result, barley stands out from other crop plants because of its crucial role in both agriculture and science. Advances in both aspects create a positive feedback loop, which allows the barley to take a leading role in meeting the great challenges of climate change, as well as the growth of the human population.

The research has shown the possibility of the existence of complex senescence regulatory mechanisms including RNA modifications (Sobieszczuk-Nowicka et al., 2018); therefore, investigation of the epigenetic mechanisms participating in leaf senescence is an important research topic. As has already been mentioned the role of m^6^A modifications in the aging of animal models as well as cell senescence has been previously reported (Castro-Hernández et al., 2022; Sun et al., 2022b). Recent findings about m^6^A modification in the process of aging include the participation of this modification in cell senescence, inflammation, autophagy, DNA damage, oxidative stress, neurodegenerative diseases, tumors, diabetes, and cardiovascular diseases. Shafik et al. reported dynamic changes in m^6^A modification sites with increasing age in the brains of mice and humans (Shafik et al., 2021). It is postulated that dynamic and reversible m^6^A modification may bring about a new target for anti-aging therapies. Nonetheless, there is no such data about the process of leaf senescence in plants. In this scope, the prospect of finding connections between the process of senescence and m^6^A modification of RNA in plants seems to be compelling.

Methylomes of differentially expressed genes during senescence in barley leaves is an interesting yet undiscovered field to research. Recent studies done on barley roots under cadmium stress involving transcriptome-wide m^6^A methylation profiles have shown a strong correlation between the m^6^A methylomes and transcriptome profiles induced by Cd stress (Su et al., 2022). Further, m^6^A transcriptome-wide analyses of wheat plants (Triticum aestivum) have shown that genes connected to plant defense are characterized by significant changes in their m6A transcript level during the response to the wheat yellow mosaic virus (WYMV) (Zhang et al., 2021; Yue et al., 2022). In contrast to a prior study (Zhou et al., 2019) the novel results demonstrated a positive association between m^6^A modification and mRNA abundance during the expansion of tomato fruits (Hu et al., 2022a). Moreover, recent findings demonstrate a vital function of mRNA methylation in Arabidopsis’s response to salt stress and reveal a close relationship between m^6^A methylation, 3’-UTR length, and mRNA stability in plant stress adaption (Hu et al., 2021).

A database-supported approach can greatly aid the course of research. Mammalian RNA modifications are accessible in many databases (MethylTranscriptome DataBase, RMBase, m^6^A 2Target, MeTDiff), in contrast with plant RNA modifications, which can be found only in one database: PRMdb (Ma et al., 2020). The gathered data are based on the analysis of thousands of RNA-seq, degradome-seq, and small RNA-seq data from a broad range of plant species with the use of the tool HAMR (high-throughput analysis of modified ribonucleotide). In the case of barley, this database describes only 10 modifications of tRNA and 10 modifications of mRNA, and there is no mention of m^6^A among them, indicating an incomplete data set, so far. Furthermore, numerous unique bioinformatic tools, such m^6^a boost and DeepPromise, have been created to look for and identify the RRACH motif surrounding m6A methylation sites in mammals, while crop species have only one weakly supervised model: m^6^A-Maize, for computational identification of m^6^A-containing regions in plants, based on high-throughput sequencing analysis and results (Liang et al., 2022).

In consideration of the fact that RNA modification m^6^A was shown to be deposited onto chromatin-associated regulatory RNA (carRNA), including RNA transcribed from transposable elements (repeat RNA) (Romano et al., 2018), the barley genome, consisting of 80% of repetitive elements, presents huge potential for manipulation. Mechanisms that show the involvement of repeat RNA in chromatin remodeling and gene transcription have been recorded in mammals (Trigiante et al., 2021). Also, m^6^A was shown to stimulate the degradation of a subset of repeat RNA species through nuclear exosome-targeting-mediated nuclear degradation (Liu et al., 2020).

Innovatory research was performed by Yu (2021), involving human FTO – an enzyme mediating RNA m^6^A demethylation and originally identified as a fat mass- and obesity-associated protein (Frayling et al., 2007) – which was introduced to the genomes of rice and potato. In greenhouse conditions, rice with transgenic FTO expression increased grain production by nearly 300%. Transgenic expression of FTO in rice and potato during field tests increased yield and biomass by about 50%. FTO has no effect on mature cell size, cell proliferation, shoot meristem, root diameter, plant height, or ploidy. However, it does stimulate the production of tiller buds and root meristem cell proliferation, as well as photosynthetic performance and tolerance to drought (Yu et al., 2021). According to the scientists, modifying plant RNA m^6^A methylation is a promising method for significantly enhancing plant growth and agricultural productivity.

Senescing cells must retain their ability to maintain homeostasis at each stage, which is why the effectiveness of the senescence process’ regulation is a sign of the vitality of the senescing cells. We have defined a decisive moment in the DILS model (Sobieszczuk-Nowicka et al., 2018) which determines the point of no return, but the mechanism of its control is yet to be uncovered. This indicates the possibility of an unknown additional switch between cell survival and cell death. Discoveries of m^6^A RNA modification changes in certain RNA species in different stages of leaf senescence may uncover the role of such modifications in the metabolic reprogramming of leaf cells between strategies of cell survival and cell death.

The consequence and abundance of m^6^A modification are thought to be caused by a dynamic interplay between RNA methyltransferases, RNA demethylases, and proteins interacting with modified RNA (Sun et al., 2021). Recent research has demonstrated that the activity of ‘writers’, ‘readers’, and ‘erasers’ is vital for plant growth and abiotic stress responses (Hu et al., 2019; Reichel et al., 2019) even though our understanding of the roles of such factors in plants is far behind that for their animal counterparts. Only recently, putative RNA m 6A reader- SlYTH1 has been shown to be involved in regulating multiple physiological processes in tomato, including seed germination, biosynthesis of gibberellin and seedling root formation (Yin et al., 2022).

It has been shown (Sobieszczuk-Nowicka et al., 2018) that the specific gene expression changes for a group of ‘writers’ in response to senescence induced by darkness. This information may suggest the regulatory role of ‘writers’, ‘readers’, and ‘erasers’ in controlling senescence processes by affecting the ratio of modification of m6A RNA. Similar upregulation of several ‘writers’ genes has been already observed during salt stress in Arabidopsis (Hu et al., 2021), while drought stress and cucumber green mottle mosaic virus (CGMMV) infection induced expression of m6A erasers in sea buckthorn and watermelon, respectively (He et al., 2021; Zhang et al., 2021).

The gene expression microarrays of barley plants exposed to dark induced senescence (Sobieszczuk-Nowicka et al., 2018) have been screened and these data identify a group of genes involved in RNA methylation processes. We focused on genes involved in RNA methylation (RNA methyltransferases, ‘writers’). **Table 1** shows six RNA methyltransferases from the group of the selected genes with their isoforms, specifically upregulated in reaction to DILS (but not developmental senescence, DLS). The identified upregulated genes include RNA methyltransferases of different RNA types, namely, enzymes modifying mRNA, tRNA, and rRNA (**Table 1**). Among them, the level of the subunit of the ‘writer’ complex, encoded by the HORVU.MOREX.r3.6HG0603200.1 gene was also significantly elevated during DILS. Although the direct effect of DILS on m6A RNA status is not clarified yet, the stress- related upregulation of ‘writers’ genes has been previously shown to increase the global m6A level, inducing m6A deposition in the 5’- and 3’- UTRs (Hu et al., 2021). As m6A located in the 3’-UTR region and near to the stop codon are involved in regulating transcript stability and transcriptome integrity (2021; Pontier et al., 2019; Zhou et al., 2019; Hou et al., 2021), as well as m6A presence in the 5’- UTR effects translation (Hou et al., 2022; Hu et al., 2022b), m6A RNA modifications are the plausible mechanism to regulate the fate and function of senescence- induced RNAs. This discovery implies the possibility of complex regulatory events in the DILS process in terms of m^6^A RNA modifications. Additionally, it suggests the importance of studying RNA methylation on different RNA species during senescence in plants.

**Table 1:** RNA methyltransferases (‘writers’) genes shown to be selectively upregulated in response to dark-induced leaf senescence (DILS) in a microarray gene expression experiment (Sobieszczuk-Nowicka et al., 2018).

	Gene ID (GenBank, Ensembl)	Description	GO – Molecular function
1	DN182156, HORVU.MOREX.r3.1HG0093780.1	RNA cap guanine-N2 methyltransferase domain containing protein, expressed	methyltransferase activity
2	TA31243_4513 HORVU.MOREX.r3.6HG0630670.1	rRNA 2-O-methyltransferase fibrillarin 2, putative, expressed	methyltransferase activity
3	TA31243_4513 HORVU.MOREX.r3.7HG0738150.1	rRNA 2-O-methyltransferase fibrillarin 2, putative, expressed	methyltransferase activity
4	TA44582_4513 HORVU.MOREX.r3.6HG0611090.1	RNA methyltransferase domain-containing protein 2, putative, expressed	no GO annotation is available
5	AK249886 HORVU.MOREX.r3.4HG0388500.1	N-dimethylguanosine tRNA methyltransferase, putative, expressed	tRNA (guanine-N2-)-methyltransferase
^6^	AK249886 HORVU.MOREX.r3.2HG0111990.1	N-dimethylguanosine tRNA methyltransferase, putative, expressed	tRNA (guanine-N2-)-methyltransferase
7	TA54202_4513 HORVU.MOREX.r3.6HG0603200.1	MT-A70 domain-containing protein, m6A RNA ‘writer’, expressed	methyltransferase activity
8	AM039903 HORVU.MOREX.r3.6HG0571740.1	RNA methyltransferase protein, putative, expressed	methyltransferase activity
9	AM039903 HORVU.MOREX.r3.6HG0571750.1	RNA methyltransferase protein, putative, expressed	methyltransferase activity
10	AM039903 HORVU.MOREX.r3.5HG0486930.1	RNA methyltransferase protein, putative, expressed	methyltransferase activity

GO, gene ontology.

## 5 Conclusions

Agriculture and crop production are significantly impacted by crop losses brought on by climate change and other abiotic stressors, factors that have an impact on how sustainably food is produced. Some of the most significant stressors include salinity, drought, and extreme temperatures (heat, cold, or freezing). All of these stressors individually impose unique complexity on a plant’s biological and genetic response, as well as on solutions to reduce them. In order to facilitate translational research, it is crucial to have a thorough understanding of the diverse response(s) to abiotic stresses and define them for each agricultural plant. Numerous reviews have concentrated on several aspects of plant abiotic stresses and outlined the need to define the processes that occur as well as possible mitigation strategies, such as the use of “antistress effectors,” the development of novel genetic approaches, and the development of new resilient crops. Decoding the molecular mechanics of development and stress resistance is crucial for better crop management, increasing barley production and quality. In plants, the improvement of crop quality by m6A editing is believed to be a promising strategy. The m6A modification demonstrated a high level of sensitivity and complexity in response to environmental changes. Importantly, crop resistance may be improved by editing m6A modifications on transcripts relevant to plant resistance (Zheng et al., 2020). There are several different RNA metabolic processes that are connected to the m6A-mediated control of gene expression. It has also been shown that this modification influences the fate of diverse RNA species including tRNAs, rRNAs, sncRNAs, lncRNAs, and micro-RNAs. Even though the significance and cellular role of m6A in different RNA types in the response of plants to environmental stimuli have been already implied (Su et al., 2022), the specific roles of these modifications are still to a large extent unknown.

## Data availability statement

Publicly available datasets were analyzed in this study. This data can be found here: GenBank (RRID: SCR_002760), Ensembl (RRID: SCR_002344).

## Author contributions

ES-N conceived the topic of the manuscript. ER, MG, MA-J, JM, UT and ES-N wrote the manuscript. MG prepared the figure. ER was responsible for the layout of the manuscript and prepared the table and the data contained therein. ES-N coordinated the writing of the manuscript. All authors listed have made a substantial, direct, and intellectual contribution to the work, and approved it for publication.

## References

[B1] Anderson S. J. Kramer M. C. Gosai S. J. Yu X. Vandivier L. E. Nelson A. D. L.. (2018). N6-methyladenosine inhibits local ribonucleolytic cleavage to stabilize mRNAs in arabidopsis. Cell Rep. 25 (5), 1146–1157.e3. doi: 10.1016/j.celrep.2018.10.020 30380407

[B2] Arribas-Hernández L. Bressendorff S. Hansen M. H. Poulsen C. Erdmann S. Brodersen P. (2018). An m^6^A-YTH module controls developmental timing and morphogenesis in arabidopsis. Plant Cell 30 (5), 952–967. doi: 10.1105/tpc.17.00833 29643069 PMC6002192

[B3] Batista P. J. Molinie B. Wang J. Qu K. Zhang J. Li L.. (2014). M^6^A RNA modification controls cell fate transition in mammalian embryonic stem cells. Cell Stem Cell 15 (6), 707–719. doi: 10.1016/j.stem.2014.09.019 25456834 PMC4278749

[B4] Beier S. Himmelbach A. Colmsee C. Zhang X. Q. Barrero R. A. Zhang Q.. (2017). Construction of a map-based reference genome sequence for barley, hordeum vulgare l. Sci. Data 4, 170044. doi: 10.1038/sdata.2017.44 28448065 PMC5407242

[B5] Breeze E. Harrison E. McHattie S. Hughes L. Hickman R. Hill C.. (2011). High-resolution temporal profiling of transcripts during arabidopsis leaf senescence reveals a distinct chronology of processes and regulation. Plant Cell 23 (3), 873–894. doi: 10.1105/tpc.111.083345 21447789 PMC3082270

[B6] Buchanan-Wollaston V. Earl S. Harrison E. Mathas E. Navabpour S. Page T.. (2003). The molecular analysis of leaf senescence-a genomics approach. Plant Biotechnol. J. 1, 3–22. doi: 10.1046/j.1467-7652.2003.00004.x 17147676

[B7] Castro-Hernández R. Berulava T. Metelova M. Epple R. Centeno T. P. Sakib S.. (2022). Conserved reduction of m6A marks during aging and neurodegeneration is linked to altered translation of synaptic transcripts. bioRxiv 2022.06.08.495100. doi: 10.1101/2022.06.08.495100

[B8] Chang Y. N. Zhu C. Jiang J. Zhang H. Zhu J. K. Duan C. G. (2020). Epigenetic regulation in plant abiotic stress responses. J. Integr. Plant Biol. 62 (5), 563–580. doi: 10.1111/jipb.12901 31872527

[B9] Chmielowska-Bąk J. Arasimowicz-Jelonek M. Deckert J. (2019). In search of the mRNA modification landscape in plants. BMC Plant Biol. 19 (1), 421. doi: 10.1186/s12870-019-2033-2 31610789 PMC6791028

[B10] Cohn W. E. (1959). 5-ribosyl uracil, a carbon-carbon ribofuranosyl nucleoside in ribonucleic acids. Biochim. Biophys. Acta 32, 569–571. doi: 10.1016/0006-3002(59)90644-4 13811055

[B11] Davis F. F. Allen F. W. (1957). Ribonucleic acids from yeast which contain a fifth nucleotide. J. Biol. Chem. 227 (2), 907–915. doi: 10.1016/s0021-9258(18)70770-9 13463012

[B12] Dominissini D. Nachtergaele S. Moshitch-Moshkovitz S. Peer E. Kol N. Ben-Haim M. S.. (2016). The dynamic N1 -methyladenosine methylome in eukaryotic messenger RNA. Nature 530 (7591), 441–446. doi: 10.1038/nature16998 26863196 PMC4842015

[B13] Eriksson M. C. Szukala A. Tian B. Paun O. (2020). Current research frontiers in plant epigenetics: an introduction to a virtual issue. New Phytol. 226 (2), 285–288. 32180259 10.1111/nph.16493 PMC7154677

[B14] Frayling T. M. Timpson N. J. Weedon M. N. Zeggini E. Freathy R. M. Lindgren C. M.. (2007). A common variant in the FTO gene is associated with body mass index and predisposes to childhood and adult obesity. Science 316 (5826), 889–894. doi: 10.1126/science.1141634 17434869 PMC2646098

[B15] Gilbert W. (1986). Origin of life: The RNA world. Nature 319, 618. doi: 10.1038/319618a0

[B16] Giraldo P. Benavente E. Manzano-Agugliaro F. Gimenez E. (2019). Worldwide research trends on wheat and barley: A bibliometric comparative analysis. Agronomy 9 (7), 352. doi: 10.3390/agronomy9070352

[B17] Gregersen P. L. Holm P. B. Krupinska K. (2008). Leaf senescence and nutrient remobilisation in barley and wheat. Plant Biol. 10 (SUPPL.1), 37–49. doi: 10.1111/j.1438-8677.2008.00114.x 18721310

[B18] Guo J. Zheng J. Zhang H. Tong J. (2022). RNA m6A methylation regulators in ovarian cancer. Cancer Cell Int. 21, 609. doi: 10.1186/s12935-021-02318-8 PMC8600856 34794452

[B19] He Y. Li L. Yao Y. Li Y. Zhang H. Fan M. (2021). Transcriptome-wide N6-methyladenosine (m6A) methylation in watermelon under CGMMV infection. BMC Plant Biol. 21, 516. doi: 10.1186/s12870-021-03289-8 34749644 PMC8574010

[B20] Hou Y. Sun J. Wu B. Gao Y. Nie H. Nie Z.. (2021). CPSF30-l-mediated recognition of mRNA m6A modification controls alternative polyadenylation of nitrate signaling-related gene transcripts in arabidopsis. Mol. Plant 14, 688–699. doi: 10.1016/j.molp.2021.01.013 33515769

[B21] Hou N. Li C. He J. Liu Y. Yu S. Malnoy M.. (2022). MdMTA-mediated m6A modification enhances drought tolerance by promoting mRNA stability and translation efficiency of genes involved in lignin deposition and oxidative stress. New Phytol. 234 (4), 1294–1314. 35246985 10.1111/nph.18069

[B22] Hu J. Cai J. Park S. J. Lee K. Li Y. Chen Y.. (2021). N6-methyladenosine mRNA methylation is important for salt stress tolerance in arabidopsis. Plant J. 106 (6), 1759–1775. doi: 10.1111/tpj.15270 33843075

[B23] Hu J. Cai J. Umme A. Chen Y. Xu T. Kang H. (2022a). Unique features of mRNA m6A methylomes during expansion of tomato (Solanum lycopersicum) fruits. Plant Physiol. 188 (4), 2215–2227. doi: 10.1093/plphys/kiab509 34730815 PMC8968293

[B24] Hu J. Cai J. Xu T. Kang H. (2022b). “Epitranscriptomic mRNA modifications governing plant stress responses: underlying mechanism and potential application,” in Plant biotechnology journal (John Wiley and Sons Inc). doi: 10.1111/pbi.13913 PMC9674322 36002976

[B25] Hu J. Manduzio S. Kang H. (2019). Epitranscriptomic RNA methylation in plant development and abiotic stress responses. Front. Plant Sci. 10. doi: 10.3389/fpls.2019.00500 PMC6499213 31110512

[B26] Jing H.-C. Hille J. Dijkwel P. P. (2003). Ageing in plants: Conserved strategies and novel pathways. Plant Biol. 5, 455–464. doi: 10.1055/s-2003-44779

[B27] Kumar S. Mohapatra T. (2021). Deciphering epitranscriptome: Modification of mRNA bases provides a new perspective for post-transcriptional regulation of gene expression. Front. Cell Dev. Biol. 9. doi: 10.3389/fcell.2021.628415 PMC8010680 33816473

[B28] Liang Z. Zhang L. Chen H. Huang D. Song B. (2022). m^6^A-maize: Weakly supervised prediction of m^6^A-carrying transcripts and m^6^A-affecting mutations in maize (Zea mays). Methods 203, 226–232. doi: 10.1016/j.ymeth.2021.11.010 34843978

[B29] Liebsch D. Keech O. (2016). Dark-induced leaf senescence: new insights into a complex light-dependent regulatory pathway. New Phytol. 212 (3), 563–570). doi: 10.1111/nph.14217 27716940

[B30] Li S. Mason C. E. (2014). The pivotal regulatory landscape of RNA modifications. Annu. Rev. Genomics Hum. Genet. 15, 127–150. doi: 10.1146/annurev-genom-090413-025405 24898039

[B31] Liu J. Dou X. Chen C. Chen C. Liu C. Michelle Xu M.. (2020). N6-methyladenosine of chromosome-associated regulatory RNA regulates chromatin state and transcription. Science 367 (6477), 580–586. doi: 10.1126/science.aay6018 31949099 PMC7213019

[B32] Ma X. Si F. Liu X. Luan W. (2020). PRMdb: A repository of predicted RNA modifications in plants. Plant Cell Physiol. 61 (6), 1213–1222. doi: 10.1093/pcp/pcaa042 32542382

[B33] Meier J. C. Kankowski S. Krestel H. Hetsch F. (2016). RNA Editing–systemic relevance and clue to disease mechanisms? Front. Mol. Neurosci. 9 (NOV2016). doi: 10.3389/fnmol.2016.00124 PMC5120146 27932948

[B34] Meyer A. J. Garry D. J. Hall B. Byrom M. M. McDonald H. G. Yang X.. (2015). Transcription yield of fully 2’-modified RNA can be increased by the addition of thermostabilizing mutations to T7 RNA polymerase mutants. Nucleic Acids Res. 43 (15), 7480–7488. doi: 10.1093/nar/gkv734 26209133 PMC4551944

[B35] Meyer K. D. Jaffrey S. R. (2014). The dynamic epitranscriptome: N6-methyladenosine and gene expression control. Nat. Rev. Mol. Cell Biol. 15 (5), 313–326). doi: 10.1038/nrm3785 24713629 PMC4393108

[B36] Miryeganeh M. (2021). Plants’ Epigenetic Mechanisms and Abiotic Stress. Genes 12, 1106. doi: 10.3390/genes12081106 34440280 PMC8394019

[B37] Paluch-Lubawa E. Stolarska E. Sobieszczuk-Nowicka E. (2021). Dark-induced barley leaf senescence – a crop system for studying senescence and autophagy mechanisms. Front. Plant Sci. 12. doi: 10.3389/fpls.2021.635619 PMC8005711 33790925

[B38] Pontier D. Picart C. El Baidouri M. Roudier F. Xu T. Lahmy S.. (2019). The m6A pathway protects the transcriptome integrity by restricting RNA chimera formation in plants. Life Sci. Alliance 2, e201900393. doi: 10.26508/lsa.201900393 31142640 PMC6545605

[B39] Reichel M. Köster T. Staiger D. (2019). Marking RNA: M^6^A writers, readers, and functions in arabidopsis. J. Mol. Cell Biol. 11 (10), 899–910). doi: 10.1093/jmcb/mjz085 31336387 PMC6884701

[B40] Romano G. Veneziano D. Nigita G. Nana-Sinkam S. P. (2018). RNA Methylation in ncRNA: Classes, detection, and molecular associations. Front. Genet. 9 (JUL). doi: 10.3389/fgene.2018.00243 PMC6052889 30050561

[B41] Růžička K. Zhang M. Campilho A. Bodi Z. Kashif M. Saleh M.. (2017). Identification of factors required for m^6^A mRNA methylation in arabidopsis reveals a role for the conserved E3 ubiquitin ligase HAKAI. New Phytol. 215 (1), 157–172. doi: 10.1111/nph.14586 28503769 PMC5488176

[B42] Saletore Y. Chen-Kiang S. Mason C. E. (2013). Novel RNA regulatory mechanisms revealed in the epitranscriptome. RNA Biol. 10 (3), 342–346. doi: 10.4161/rna.23812 23434792 PMC3672275

[B43] Shafik A. M. Zhang F. Guo Z. Dai Q. Pajdzik K. Li Y.. (2021). N6-methyladenosine dynamics in neurodevelopment and aging, and its potential role in alzheimer’s disease. Genome Biol. 22 (1), 17. doi: 10.1186/s13059-020-02249-z 33402207 PMC7786910

[B44] Shao Y. Wong C. E. Shen L. Yu H. (2021). N6-methyladenosine modification underlies messenger RNA metabolism and plant development. Curr. Opin. Plant Biol. 63, 102047. doi: 10.1016/j.pbi.2021.102047 33965696

[B45] Shen M. Li Y. Wang Y. Shao J. Zhang F. Yin G.. (2021). N6-methyladenosine modification regulates ferroptosis through autophagy signaling pathway in hepatic stellate cells. Redox Biol. 47, 102151. doi: 10.1016/j.redox.2021.102151 34607160 PMC8495178

[B46] Sobieszczuk-Nowicka E. Wrzesiński T. Bagniewska-Zadworna A. Kubala S. Rucińska-Sobkowiak R. Polcyn W.. (2018). Physio-genetic dissection of dark-induced leaf senescence and timing its reversal in Barley1[OPEN]. Plant Physiol. 178 (2), 654–671. doi: 10.1104/PP.18.00516 30126868 PMC6181038

[B47] Sokpor G. Nguyen H. P. Tuoc T. (2021). Context-specific chromatin remodeling activity of mSWI/SNF complexes depends on the epigenetic landscape. Signal Transduct. Target. Ther. 6 (1), 360. doi: 10.1038/s41392-021-00770-6 34615852 PMC8494729

[B48] Su T. Fu L. Kuang L. Chen D. Zhang G. Shen Q.. (2022). Transcriptome-wide m^6^A methylation profile reveals regulatory networks in roots of barley under cadmium stress. J. Hazard. Mater. 423, 127140. doi: 10.1016/j.jhazmat.2021.127140 34523471

[B49] Sun C. Ali K. Yan K. Fiaz S. Dormatey R. Bi Z.. (2021). Exploration of epigenetics for improvement of drought and other stress resistance in crops: A review. Plants 10 (6), 1226. doi: 10.3390/plants10061226 34208642 PMC8235456

[B50] Sun B. Bhati K.K. Edwards A. Petri L. Blaakmeer A. Dolde U. Rodrigues V.. (2022a). The m 6 a writer FIONA1 methylates the 3’UTR of FLC and controls 2 flowering in arabidopsis. bioRxiv, 2022.01.24.477497. doi: 10.1101/2022.01.24.477497

[B51] Sun J. Cheng B. Su Y. Li M. Ma S. Zhang Y.. (2022b). The potential role of m^6^A RNA methylation in the aging process and aging-associated diseases. Front. Genet. 13, 869950. doi: 10.3389/fgene.2022.869950 35518355 PMC9065606

[B52] Thomas H. (2013). Senescence, ageing and death of the whole plant. New Phytol. 197 (3), 696–711). doi: 10.1111/nph.12047 23176101

[B53] Trigiante G. Blanes Ruiz N. Cerase A. (2021). Emerging roles of repetitive and repeat-containing RNA in nuclear and chromatin organization and gene expression. Front. Cell Dev. Biol. 9. doi: 10.3389/fcell.2021.735527 PMC8552494 34722514

[B54] Wang X. Jiang B. Gu L. Chen Y. Mora M. Zhu M.. (2021). A photoregulatory mechanism of the circadian clock in arabidopsis. Nat. Plants 7 (10), 1397–1408. doi: 10.1038/s41477-021-01002-z 34650267

[B55] Wang S. Lv W. Li T. Zhang S. Wang H. Li X.. (2022). Dynamic regulation and functions of mRNA m^6^A modification. Cancer Cell Int. 22 (1), 48. doi: 10.1186/s12935-022-02452-x 35093087 PMC8800407

[B56] Williams G. D. Gokhale N. S. Horner S. M. (2019). Regulation of viral infection by the RNA modification N6-methyladenosine. Annu. Rev. Virol. 6, 17–18. doi: 10.1146/annurev-virology-092818 PMC6884077 31283446

[B57] Wu Z. Wang S. Belmonte J. C. I. Zhang W. Qu J. Liu G.-H. (2022). Emerging role of RNA m^6^A modification in aging regulation. Curr. Med. 1 (1), 8. doi: 10.1007/s44194-022-00009-8

[B58] Yang Y. Hsu P. J. Chen Y. S. Yang Y. G. (2018). Dynamic transcriptomic m^6^A decoration: Writers, erasers, readers and functions in RNA metabolism. Cell Res. 28 (6), 616–624). doi: 10.1038/s41422-018-0040-8 29789545 PMC5993786

[B59] Yin S. Ao Q. Qiu T. Tan C. Tu Y. Kuang T.. (2022). Tomato SlYTH1 encoding a putative RNA m^6^A reader affects plant growth and fruit shape. Plant Sci. 323, 111417. doi: 10.1016/j.plantsci.2022.111417 35973580

[B60] Yue J. Wei Y. Zhao M. (2022). The reversible methylation of m^6^A is involved in plant virus infection. Biology 11 (2), 271. doi: 10.3390/biology11020271 35205137 PMC8869485

[B61] Yu Q. Liu S. Yu L. Xiao Y. Zhang S. Wang X.. (2021). RNA Demethylation increases the yield and biomass of rice and potato plants in field trials. Nat. Biotechnol. 39 (12), 1581–1588. doi: 10.1038/s41587-021-00982-9 34294912

[B62] Yu X. Sharma B. Gregory B. D. (2021). The impact of epitranscriptomic marks on post-transcriptional regulation in plants. Briefings Funct. Genomics 20 (2), 113–124. doi: 10.1093/bfgp/elaa021 33274735

[B63] Zaccara S. Ries R. J. Jaffrey S. R. (2019). Reading, writing and erasing mRNA methylation. Nat. Rev. Mol. Cell Biol. 20 (10), 608–624). doi: 10.1038/s41580-019-0168-5 31520073

[B64] Zhang T. Y. Wang Z. Q. Hu H. C. Chen Z. Q. Liu P. Gao S. Q.. (2021). Transcriptome-wide N6-methyladenosine (m^6^A) profiling of susceptible and resistant wheat varieties reveals the involvement of variety-specific m^6^A modification involved in virus-host interaction pathways. Front. Microbiol. 12. doi: 10.3389/fmicb.2021.656302 PMC8187603 34122371

[B65] Zheng H.X. Sun X. Zhang X.S. Sui N. (2020). m^6^A editing: New tool to improve crop quality? Trends Plant Sci. 25 (9), 859–867). doi: 10.1016/j.tplants.2020.04.005 32376086

[B66] Zhou L. Gao G. Tang R. Wang W. Wang Y. Tian S.. (2022). m^6^A-mediated regulation of crop development and stress responses. Plant Biotechnol. J. 20, 1447–1455. doi: 10.1111/pbi.13792 35178842 PMC9342612

[B67] Zhou L. Tang R. Li X. Tian S. Li B. Qin G. (2021). N6-methyladenosine RNA modification regulates strawberry fruit ripening in an ABA-dependent manner. Genome Biol. 22, 168. doi: 10.1186/s13059-021-02385-0 34078442 PMC8173835

[B68] Zhou L. Tian S. Qin G. (2019). RNA Methylomes reveal the m6A-mediated regulation of DNA demethylase gene SlDML2 in tomato fruit ripening. Genome Biol. 20 (1), 156. doi: 10.1186/s13059-019-1771-7 31387610 PMC6683476

[B69] Zimmermann P. Heinlein C. Orendi G. Zentgraf U. (2006). Senescence-specific regulation of catalases in arabidopsis thaliana (L.) heynh. Plant Cell Environ. 29 (6), 1049–1060. doi: 10.1111/j.1365-3040.2005.01459.x 17080932

